# Fatal Ovarian Hemorrhage Associated With Anticoagulation Therapy in a Yucatan Mini-Pig Following Venous Stent Implantation

**DOI:** 10.3389/fvets.2020.00018

**Published:** 2020-01-30

**Authors:** Sophie Boorman, Hope Douglas, Bernd Driessen, Matthew J. Gillespie, Thomas P. Schaer

**Affiliations:** ^1^Department of Clinical Sciences, College of Veterinary Medicine, JT Vaughan Large Animal Teaching Hospital, Auburn University, Auburn, AL, United States; ^2^Department of Clinical Studies, New Bolton Center, School of Veterinary Medicine, University of Pennsylvania, Philadelphia, PA, United States; ^3^Department of Cardiology, The Children's Hospital of Philadelphia, Philadelphia, PA, United States

**Keywords:** endovascular, ovary, animal model, pre-clinical, bleed

## Abstract

Swine models are commonly utilized in endovascular research for development of intravascular interventions and medical device development. As part of a pilot study for a venous vascular stent device, a 5-year-old female Yucatan mini-pig underwent bilateral external iliac vein stent placement under general anesthesia. To reduce thrombotic complications by reduction of thrombus formation on wires, sheaths, and catheters, the pig was heparinized with a total of 300 IU/kg of heparin, establishing an activated clotting time (ACT) of 436 s. The ACT had returned to below 200 s by the end of the procedure. To prevent postoperative thrombosis, the pig received an anticoagulation therapy protocol consisting of enoxaparin, clopidogrel, and aspirin. There were no complications during the immediate postoperative period. However, the pig died 4 days after surgery. Necropsy established the cause of death as abdominal exsanguination due to severe, acute, intra-ovarian hemorrhage, most likely related to ovulation. Life-threatening ovarian hemorrhage is occasionally seen in women with congenital or acquired bleeding disorders; to our knowledge this is the first report of fatal ovarian hemorrhage in an animal enrolled in a pre-clinical research trial.

## Background

The development of minimally invasive surgical techniques and devices has facilitated the rapid expansion of the field of endovascular medicine (1). Specifically, the treatment of peripheral vascular diseases has been revolutionized by percutaneous endovascular interventions such as angioplasty and stent placement. The necessity for careful anticoagulant therapy during these procedures to prevent life-threatening thrombosis, and to maintain the patency of positioned endovascular devices, has been well-established (2). Pre-clinical animal trials, in particular those utilizing large animal models, play a crucial role during product development and in regulation of these technologies, establishing the safety of future devices before progression to human studies (3). The pig has become the preferred model for studying certain intravascular devices (4) owing in part to similarities in the cardiovascular anatomy, physiology, and histology between pigs and humans (5–8), as well as avoiding the intensified ethical concerns associated with the use of non-human primates.

In human medicine, the rupture of the corpus luteum during normal ovulation may rarely lead to life-threatening hemorrhage into the peritoneal cavity (9). Women most at risk are those with congenital or acquired bleeding disorders, or those treated with anticoagulants for thrombotic conditions (10). To the author's knowledge, this condition has not been described in the sow. We describe here a case of fatal intra-abdominal exsanguination originating from the ovary in a sow that underwent bilateral external iliac vein stent implantation. The sow was part of a pre-clinical pilot trial for a novel endovascular stent; the implications for endovascular research are discussed.

## Case Presentation

A 5-year old female intact Yucatan mini-pig (obtained aged 1.5 years from BioSinclair Resources, Auxvasse, Missouri, USA), part of a resident colony, was enrolled into this pilot study. The aim of this study was to gain first impressions of device handling, deployment and early *in vivo* performance of a self-expanding stent with an inner polymeric valve, designed to be delivered intravascular by a catheter-based delivery-system. The pig was recruited from our resident colony to facilitate the rapid generation of initial pilot data to progress the study quickly past the proof-of-concept stage. The enrolled naïve animal had no history of any medical abnormalities and weighed 66 kg at the time of surgery. All animal procedures were performed at the Preclinical Translational Core of the Large Animal Hospital, New Bolton Center, School of Veterinary Medicine, University of Pennsylvania, with approval from the Institutional Animal Care and Use Committee (IACUC Protocol number 804541).

Several months prior to the stent implantation surgery, the pig underwent a computed tomographic (CT) scan of the caudal abdomen and inguinal region to obtain measurements of the right and left external iliac veins at the level of the target region for the planned implantation. These measurements were intended to guide the device engineers when sizing and manufacturing the stents. Treatment with enoxaparin (2 mg/kg subcutaneous (SQ) q 12 h, Sandoz, Inc., Holzkirchen, Germany) (11), clopidogrel (75 mg per os (PO) q 24 h, Aurobindo Pharma., New Jersey, USA), and aspirin (81 mg PO q 24 h, Major Pharmaceuticals, Livonia, Michigan, USA) was initiated the day before surgery, which would continue for the entire post-operative period. A single dose of nifedipine (30 mg PO, Oceanside Pharmaceuticals Inc., California, USA) was also administered the day before surgery to reduce vascular spasm (12). A preoperative analgesic protocol was initiated, consisting of Buprenorphine (0.01 mg/kg intramuscular (IM) q 12 h, Ceva Animal Health, Cambridge, Ontario, Canada) and flunixin meglumine (1.1 mg/kg IM q 24 h, Merck Animal Health, Intervet Inc., Madison, New Jersey, USA) which continued throughout the postoperative period. At the day of surgery, routine preoperative physical examination, complete blood count and serum chemistry were within normal limits. A coagulation profile revealed 500 ng/ml D-Dimers, 489 mg/dl fibrinogen, 225,500 /μl platelets, a prothrombin time of 12.6 s, an activated partial thromboplastin time of 8.7 s and an antithrombin III of 83% (Instrumentation Laboratory ACL Elite Series, Bedford, Massachusetts, USA). A coagulation profile was performed in three additional healthy and naïve members of the swine colony and the results obtained were similar, suggesting that these values were within normal range for the age of these Yucatan mini-pigs.

For general anesthesia, the animal was sedated with midazolam (0.3 mg/kg, Pfizer, New York, USA), butorphanol (0.3 mg/kg, Zoetis Inc., Orion Pharma, Kalamazoo, Michigan, USA) and dexmedetomidine (0.03 mg/kg, Zoetis Inc., Orion Pharma, Kalamazoo, Michigan, USA) combined in the same syringe and administered intramuscularly. Following peripheral catheter placement into the left ear under aseptic conditions, general anesthesia was induced with 0.45 mg/kg propofol intravenous (IV - Zoetis Inc., Orion Pharma, Kalamazoo, Michigan, USA) and was maintained with isoflurane in oxygen (1.5–3%). An arterial catheter was positioned into the auricular artery, and blood pressure monitored throughout (mean arterial blood pressure ranged from 80 to 105 mmHg). For the duration of the procedure, the pig was administered a lidocaine continuous rate infusion (150 μg/kg/min loading dose administered over 15 min (total dose was 150 mg) followed by 50 μg/kg/min IV, Phoenix Pharmaceutical, Inc., St. Joseph, Missouri, USA), to reduce the inhalant anesthetic requirements. Temperature was measured by rectal thermometer at 5 min intervals and ranged from 32.2 to 36.0°C; hypothermia was prevented using a forced-air warming blanket (Bair Hugger System, 3M, St. Paul, Minnesota, USA). Following aseptic preparation of the ventral neck and both inguinal regions, the surgical sites were aseptically draped for surgery.

The right external jugular vein was identified using ultrasound (Aplio 300, Canon Medical Systems USA, California, USA), and a 16-French access sheath was placed via the Seldinger technique under ultrasonographic guidance. A baseline activated clotting time (ACT) was obtained and was 123 s (i-STAT Kaolin ACT Cartridge, Abbott Point of Care Inc., Princeton, New Jersey, USA). Once the sheath was positioned within the jugular vein, 200 IU/kg of unfractionated heparin (5,000 USP units/mL, Pfizer, New York, USA) was administered intravenously, aimed at establishing an ACT of 250–350 s to avoid post-implantation thrombi formation. Approximately 20 min following the initial dose of heparin, the activated clotting time was 220 s. In order to further increase the ACT, an additional 100 IU/kg sodium heparin were administered intravenously. Ten minutes following administration of the second dose of heparin, the ACT was 436 s.

**Figure 1:**
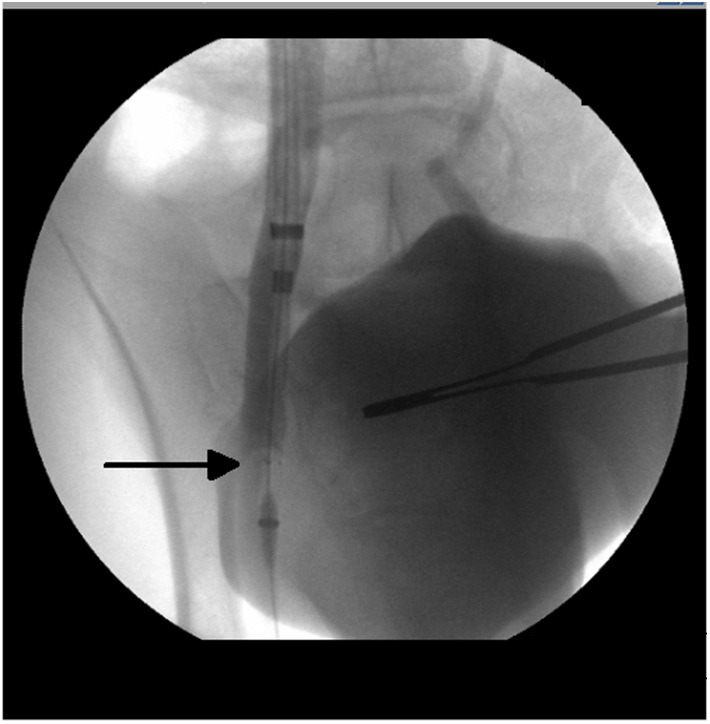
Radiographic image from a venogram performed of the right external iliac vein with the test article (venous stent with polymeric inner valve—arrow) positioned *in situ*.

**Figure 2:**
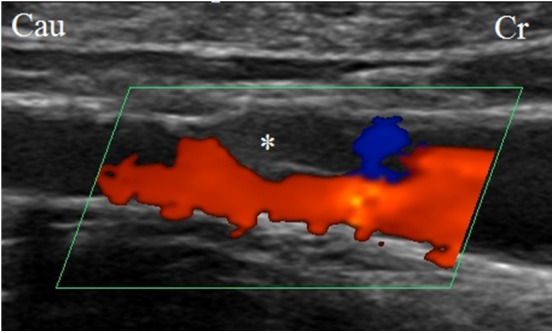
Ultrasonographic image of the right external iliac artery with the test article (venous stent with polymeric inner valve—valve is indicated by asterix) positioned *in situ*. The blood flow was unidirectional.

A venogram (100 ml Ioxhexol, GE Healthcare, Chicago, Illinois, Fluoroscope: Ziehm Vision) was performed of the targeted locations prior to the positioning of the test article (stent with valve, Figure 1) within the right external iliac vein and reference article (stent without valve) within the left external iliac vein. Transcutaneous ultrasonographic evaluation of the external iliac veins (Aplio i700, Canon Medical Systems USA, California, USA) confirmed placement of the stents at the intended locations and unidirectional blood flow through the inner valve of the test article (Figure 2). Time to bilateral stent placement was 1 h and 35 min. The ACT was measured hourly; based on previous experience, the criteria for safe sheath removal was an ACT of <200. A table of the measured ACT values for each time point is provided in Table 1. Once the ACT returned to 196 s, the introducer was removed, and the final ACT measured prior to recovery from anesthesia was 180 s. The animal recovered from general anesthesia, and the total anesthesia time (from endotracheal intubation to extubation) was 10 h and 35 min. The pig's rectal temperature at extubation was 37.8°C. The pig was active and seeking food within 20 min of extubation. Anesthesia time was impacted by the need to achieve a target activated clotting time prior to sheath removal from the jugular vein.

**Table 1:** ACT measurements recorded for a Yucatan mini-pig during venous stent implantation that later died following ovarian hemorrhage associated with anticoagulation therapy.

**Time since induction of anesthesia**	**ACT (s)**	**Action taken**
1 h	123 (baseline)	200 IU/kg heparin administered
1 h 20 min	220	100 IU/kg heparin administered
1 h 30 min	436	
2 h 20 min	425	
3 h 5 min	412	End of surgery
4 h 45 min	347	
6 h	220	
6 h 45 min	249	Rechecked and confirmed
7 h 45 min	233	
8 h 30 min	196	Removed introducer and applied manual pressure to the jugular vein for 30 min
9 h 15 min	180	Taken to recovery

No complications were observed in the immediate postoperative period, during which time the animal was housed separately from the rest of the colony. On the third postoperative day, clinical examination was unremarkable with the exception of the animal being quieter than previously observed and inappetent. Handfeeding later that day was successful. Buprenorphine (0.01 mg/kg IM), omeprazole (0.5 mg/kg PO, Glenmark Pharmaceuticals, India), flunixin meglumine (1.1 mg/kg IM), maropitant (1 mg/kg SQ, Zoetis Inc., Orion Pharma, Kalamazoo, Michigan, USA), and 1 L of water per rectum were administered. Fluids were administered per rectum as the previously positioned auricular catheter had already been removed. Attempts to obtain blood for hematologic analysis (hematology, coagulation profile, sonoclot) were unsuccessful without undue stress, and the decision was made to continue to monitor the pig's progress overnight. Regular observations (every 4 h) were unremarkable.

**Figure 3:**
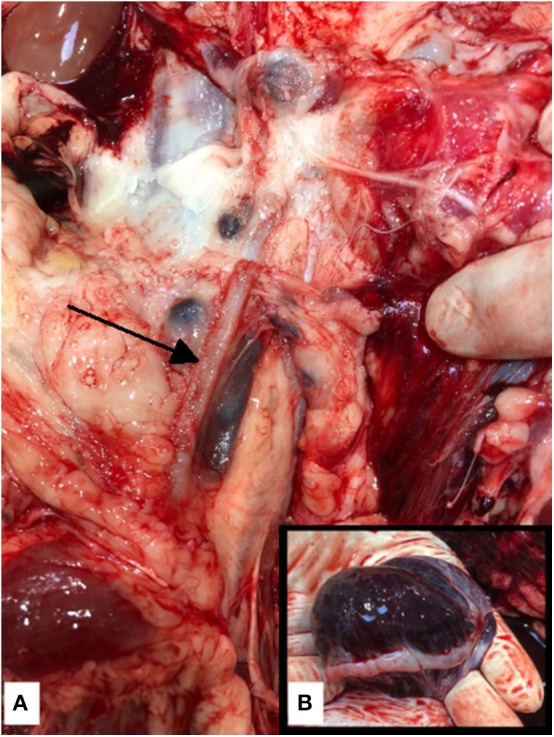
**(A)** Post mortem photograph of the left external iliac vein. The venous stents were identified in the right and left (arrow) external iliac veins without associated vessel perforation or thrombi, precluding the stents as the cause of the fatal hemoabdomen. **(B)** Post mortem photograph of the left ovary. The left ovary was expanded with blood—ovarian hemorrhage was considered to be the source of hemorrhage and thus the cause of death. The right ovary was considered to be normal in size and had pinpoint foci of hemorrhage with histological retention of the normal architecture.

On the morning of the fourth postoperative day, the animal was found dead, having last been observed 2 h prior. A necropsy performed by a board certified veterinary pathologist, identified extensive intra-abdominal hemorrhage (around 1,500 ml, equating to 30% of total blood volume) as the cause of death. Clotted blood adhered to the abdominal viscera. The right jugular vein, which was the site of the access sheath, was dissected and evaluated. Examination revealed contusions but no signs of overt hemorrhage, precluding the jugular site as the source of the fatal hemorrhage. The stents were identified within the external iliac veins, without associated hemorrhage or discernible thrombi within the vessels (Figure 3A). Patency of the stents was confirmed via visual inspection and lack of palpable thrombi within the stents. The endometrium contained numerous pin-point to 6 mm cysts containing translucent colorless fluid (histology of the uterus confirmed a diagnosis of mild cystic endometrial hyperplasia). The left ovary measured 7 × 5 × 4 cm and was expanded by clotted, friable blood, which obscured the normal ovarian architecture (Figure 3B). The right ovary measured 3 × 2 × 1.5 cm and contained pinpoint foci of hemorrhage with histological retention of the normal architecture.

Histopathologic analysis of the left ovary revealed severe acute intra-ovarian hemorrhage; the normal ovarian architecture was replaced with hemorrhage. Free erythrocytes mixed with fibrin, edema and reactive fibroblasts were identified within the tissue planes. The ovary was considered to be the source of the hemoabdomen and thus intra-abdominal exsanguination due to ovarian hemorrhage was ruled as the cause of death.

## Discussion

We describe in this report a rare postoperative complication of an uneventful endovascular procedure in a Yucatan sow. In the human patient, spontaneous bleeding during ovulation is usually inconsequential (13). Usually a rudimental amount of hemorrhage occurs when the ovary bursts from the Graafian follicle as the corpus luteum is formed; hemorrhage can also occur in cases of ovarian cyst rupture (10, 14). Occasionally, this can lead to potentially life-threatening hemoperitoneum (9). Treatment is usually conservative (15), however surgical management with oophorectomy with or without salpingectomy is also described (10, 16). Women most at risk are those with congenital or acquired coagulopathies or those taking antithrombotic medications (10, 15, 17, 18). The sow in this report had unfractionated heparin (UFH) administered intraoperatively, as well as several anticoagulant medications administered perioperatively, due to the well-known thrombogenicity associated with intravascular guidewire manipulation (19, 20). It is very likely that the animal's compromised coagulation cascade negatively impacted the ability to stop hemorrhage following the physiological event of ovulation. Additionally, the pig was hypothermic during anesthesia, which although is common for swine, which likely contributed to the coagulopathy (21). However, as histopathology of the left ovary could not confirm the precise etiology of the hemorrhage, a ruptured ovarian cyst cannot be definitively excluded. Cystic ovarian disease in swine has been reported in the literature in conjunction with uterine lesions. While cystic ovarian disease does not appear to be a very prevalent reproduction complication in mini-pigs, the development of uterine lesions has been reported to be associated with aging (22).

Heparin is a glycosaminoglycan and affects the coagulation cascade principally through an interaction with antithrombin III. The interaction of enzyme and inhibitor inactivates coagulation enzymes, namely thrombin and Xa (23). The heterogeneity of heparin in molecular size and weight, pharmacokinetic effects including coagulation explains the non-linearity heparin has on the coagulation cascade. UFH is a mixture of sulphated glycosaminoglycans of variable lengths and molecular weights. The anticoagulant effects and pharmacological properties vary with the size of the molecules and it has limited bioavailability because it binds to plasma proteins, platelets (platelet factor 4), macrophages, and endothelial cells, resulting in a highly variable anticoagulant response. UFH potentiates the effects of antithrombin III on factor Xa and thrombin (23, 24). Heparin is cleared from the blood via two principle mechanisms, and its clearance is dependent upon dosage and molecular weight. The saturable mechanism involves clearance by the reticuloendothelial system and endothelial cells. The non-saturable mechanism involves renal excretion. Administration of low doses of unfractionated heparin results in elimination primarily by the saturable mechanism. Clearance of higher doses of unfractionated heparin is predominantly by the non-saturable mechanism (25). However, the effect of bolus administration of unfractionated heparin is variable, and it can be influenced by body mass and concurrent thrombolytic drug administration (26, 27). UFH was used intra-operatively in this case for thromboprophylaxis to achieve a target ACT of 250–350 s (28, 29). The prolonged duration of inhibition of coagulation in this case, apparent in the time it took the ACT to normalize, was unexpected, even when the variable effect of heparin on coagulation is considered.

The principle components of the porcine and human coagulation system exhibit a certain degree of similarity, however some differences in the coagulation parameters and the concentration of single factors between pigs and humans exist (30). For example, several studies have shown that porcine blood has a short APTT (activated partial thromboplastin time) compared to human blood (31, 32), indicative of an accelerated intrinsic cascade in pigs. Recently, rotational thromboelastometry (ROTEM?) has emerged as a more sophisticated means of evaluating hemostasis, including providing information on clotting time, clot formation, clot stability and lysis (33). In Kessler et al. (34) compared anesthetized human and porcine blood using ROTEM® and found that while porcine clot strength was similar to human, clotting time was significantly longer, clot formation time was shorter and the clot index lysis was lower, demonstrating that initiation and propagation of clotting, as well as clot lysis, are significantly different between pigs and humans. The authors are not aware of any current studies examining the differences in heparin pharmacokinetics between humans and pigs, however, due to the relatively hypercoagulable blood of swine (35) a higher dose of heparin than that used for humans may be required, or more frequent dosing, to avoid potentially life-threatening thrombosis.

Although a higher dose of heparin may be required in porcine studies, the most important potential complication is hemorrhage. The overall dose of 300 IU/kg of heparin used in this case is not dissimilar to other porcine pre-clinical studies (36–38) and recent research has suggested that using a lower initial dose (100 IU/kg) followed by a low dose continuous rate infusion (66 IU/kg/h) may be efficient for thrombosis prophylaxis while decreasing the risk of hemorrhage (39). The heparin dosing regimen used in this case was based upon surgeon preference, extrapolated from clinical cases, and based on previous experience, though perhaps a smaller dose may have been beneficial in this case. The sow initially received a smaller dose (see Table 1) but this did not have the desired effect. Protamine has been used to neutralize the effects of heparin. However, accidental overdosing can also cause hemostatic abnormalities requiring blood transfusion (40). In addition, protamine administration has also been associated with pulmonary hypertension in pigs (36). Protamine was not available at the time of experimentation in this case. Due to the normally relatively short half-life of heparin (~1.5 h) (41), and the non-linear heparin dose-response and elimination curves, protamine treatment is not always required (42), though may have been helpful in this case.

In addition to the heparin administered intraoperatively, the sow in this report was administered anticoagulant and antithrombotic medications both pre and postoperative in an attempt to prevent thrombosis and retain implant patency. Enoxaparin is a low-molecular-weight-heparin that has been shown to be effective in treatment and prevention of deep vein thrombosis (43). Aspirin exerts its antithrombotic effect through its irreversible inhibition of platelet cyclooxygenase-1 and inhibition of thromboxane A_2_ (44). Clopidogrel is also a platelet inhibitor, though its mechanism of action is less well-defined. It may inhibit the adenosine diphosphate receptor present on platelets, preventing aggregation (45). The combination of clopidogrel and aspirin (dual-antiplatelet therapy – DAPT) in vascular surgery has been shown in randomized controlled clinical trials to provide greater inhibition of platelets in patients undergoing vascular surgery than those receiving aspirin alone (46), this inhibition theoretically greatly reduces the risk of vascular stent occlusion (47). However, multiple meta-analyses both report benefit of DAPT (48) and no benefit of DAPT (49) in vascular interventions, additionally both an increased risk of bleeding with DAPT (50) and no increased risk of bleeding with DAPT is reported (51), making recommendations for therapy in pre-clinical trials unclear. Aspirin, clopidogrel and enoxaparin combination therapy has been shown to reduce infarctions without increasing bleeding (52). Additionally, we chose this combination because of the aforementioned relative hypercoagulability of swine, as well as our increased concern for stent occlusion due to the presence of the inner polymeric valve (which may have increased blood turbulence). Although our protocol is one that has been used safely in the clinical setting, it likely contributed to our sow's inability to survive the physiological event of ovulation.

The sow described here had several cystic-like structures in the endometrium, due to cystic endometrial hyperplasia (CEH). CEH appears to be a relatively common disorder of swine (53), in a review of 298 ovariohysterectomies performed in pet pigs, 5% had lesions consistent with CEH (54). In humans, endometrial hyperplasia is associated with hyperestrogenism, such as in hormone replacement therapy or from overproduction of estrogen by fat cells in obesity (55). In swine, iatrogenic hyperestrogenism induced by experimental administration of the estrogenic metabolite zearalenone resulted in CEH (56), suggesting a similar hormonal etiology to that seen in humans. Chronic endometrial stimulation in polyestrous animals not used for breeding can lead to hyperestrogenism (22, 57); the sow in this case report was a middle-aged pig that had never farrowed. In humans, endometrial hyperplasia is associated with uterine hemorrhage, but unlikely to cause ovarian hemorrhage (58). Hyperestrogenism has the potential to alter heparin metabolism (59), increasing the half-life and bioavailability. Although we do not think that the CEH caused the ovarian hemorrhage seen in this sow, this condition may have impacted the animal's ability to manage the anticoagulation protocol, which may add a further explanation as to why our sow's activated clotting time did not return to safe levels for such a protracted period.

The sow in this report was part of a pre-clinical vascular study. Although clinically several sex-related differences in cardiovascular anatomy, pathophysiology and intervention response have been identified (60–62), sex is rarely evaluated in short-term pre-clinical animal models as a confounding variable. In 2018 Kunio et al. (63) examined the sex-related differences in the outcome of endovascular stent implantation in swine. They found that female swine had histopathological signs of vascular injury and inflammation for longer than males and that adventitial fibrosis and neointimal fibrin deposition (thickening of arterial walls and decreased arterial lumen space) were greater in males than females. These differences indicate that, just as differences between sexes are seen clinically, they may also be important at a preclinical level. Indeed, the National Institutes of Health currently urges a balanced approach to sex in pre-clinical animal models to prevent male sex-biased research from translating into adverse consequences for women's health (64). At the time of project planning, and device design, our program had a number of resident sows available to quickly assist our collaborators with sizing of the device guided by CT imaging. At the completion of device prototyping, we then enrolled the same animal into this pilot study for optimal device sizing with the target vessels. In reality, preclinical research more frequently sources younger animals for these studies, and it is rather unlikely that animals older than 2–3 years of age would be used for endovascular research in healthy test systems. Conversely, with increased interest in large animal models exhibiting comorbidities, older animals may be of interest to investigators. With increasing age, the likelihood of underlying pathologies, such as reproductive disease, increases.

One strategy of mitigating estrus cycle-related complications is to manage estrus in sows. The corpus luteum of the pig has a life span of 14–16 days and is resistant to the luteolytic action of uterine prostaglandin F2α secretion before day 12 of the estrus cycle. Shimatsu et al. showed that treatment with altrenogest, a synthetic progestogen that inhibits the release of gonadotrophin-releasing hormone, can be used to synchronize estrus and ovulation in swine (65). Treatment with 10 mg/day altrenogest could be advisable for female swine enrolled in similar vascular studies – this drug will prevent ovulation until it is withdrawn. Five to seven days after cessation of treatment, the sow will ovulate. There is some evidence in humans that progestogen-only contraceptives may increase the thrombosis risk in some individuals (66), and it is unknown if this is also true in swine. Therefore, it would be safest to schedule the surgical procedure in animals considered to be at increased risk for around 10 days after the treatment course, allowing time for the sow to ovulate prior to the iatrogenic disruption to the coagulation profile. This would also prevent any unintended disruption to the animal model.

In summary, our report describes a rare complication of anticoagulant therapy, fatal ovarian hemorrhage. The anticoagulation therapy was employed to negate the thrombotic risk associated with a catheter-based procedure in swine models. One of the limitations of this report includes the inability to definitively diagnose the ovarian histopathology (i.e., ovulation vs. ruptured cyst) due to diffuse loss of the ovarian architecture. Another is the lack of postoperative comprehensive bloodwork or a coagulation profile that may have provided more indication as to when the bleeding started. Maintenance of an intravenous catheter in swine is notoriously difficult but having venous access would have improved our postoperative monitoring. The dose of heparin provided, though not unusual, was on the high end of the range. It may be prudent for researchers to consider this rare complication associated with the physiological estrus cycle of swine when enrolling animals into studies requiring aggressive anticoagulant therapy, and to consider use of altrenogest as a safety precaution.

## Data Availability Statement

All datasets generated for this study are included in the manuscript.

## Ethics Statement

The animal study was reviewed and approved by University of Pennsylvania Institutional Animal Care and Use Committee.

## Author Contributions

SB and HD contributed to the writing of the manuscript. SB, MG, and TS managed the surgical procedure and post-operative care of the patient. HD and BD performed the general anesthesia. All authors contributed to the writing, editing, critical review of the manuscript, and gave final approval of the report.

### Conflict of Interest

The authors declare that this study received funding from Venarum Medical LLC. The funding was provided for the pilot study, this study reports an additional finding of the study rather than the primary results. The funders did not otherwise contribute to this manuscript.
